# Real‐world effectiveness of nivolumab and subsequent therapy in Japanese patients with metastatic renal cell carcinoma (POST‐NIVO study): 36‐month follow‐up results of a clinical chart review

**DOI:** 10.1111/iju.15206

**Published:** 2023-05-29

**Authors:** Junji Yonese, Nobuyuki Hinata, Satoru Masui, Yasutomo Nakai, Suguru Shirotake, Ario Takeuchi, Teruo Inamoto, Masahiro Nozawa, Kosuke Ueda, Toru Etsunaga, Takahiro Osawa, Motohide Uemura, Go Kimura, Kazuyuki Numakura, Kazutoshi Yamana, Hideaki Miyake, Satoshi Fukasawa, Naoto Morishima, Hiroaki Ito, Hirotsugu Uemura

**Affiliations:** ^1^ Department of Urology Cancer Institute Hospital of Japanese Foundation for Cancer Research Koto‐ku Tokyo Japan; ^2^ Division of Urology, Department of Surgery Related Kobe University Graduate School of Medicine Kobe Hyogo Japan; ^3^ Division of Reparative and Regenerative Medicine, Nephro‐Urologic Surgery and Andrology, Institute of Medical Life Science Mie University Graduate School of Medicine Tsu Mie Japan; ^4^ Department of Urology Osaka International Cancer Institute Osaka Japan; ^5^ Department of Uro‐Oncology Saitama Medical University International Medical Center Hidaka Saitama Japan; ^6^ Department of Urology Kyushu University Graduate School of Medical Sciences Fukuoka Japan; ^7^ Department of Urology Osaka Medical and Pharmaceutical University Takatsuki Osaka Japan; ^8^ Department of Urology Kindai University Faculty of Medicine Osakasayama Osaka Japan; ^9^ Department of Urology Kurume University School of Medicine Fukuoka Japan; ^10^ Department of Urology Isesaki Municipal Hospital Isesaki Gunma Japan; ^11^ Department of Urology Hokkaido University Graduate School of Medicine Sapporo Hokkaido Japan; ^12^ Department of Urology Osaka University Graduate School of Medicine Suita Osaka Japan; ^13^ Department of Urology Nippon Medical School Hospital Bunkyo‐ku Tokyo Japan; ^14^ Department of Urology Akita University Graduate School of Medicine Akita Japan; ^15^ Department of Urology, Molecular Oncology Niigata University Graduate School of Medical and Dental Sciences Niigata Japan; ^16^ Department of Urology Hamamatsu University School of Medicine Hamamatsu Shizuoka Japan; ^17^ Prostate Center and Division of Urology Chiba Cancer Center Chiba Japan; ^18^ Oncology Medical Affairs Ono Pharmaceutical Co., Ltd. Osaka Japan; ^19^ Oncology Medical Bristol‐Myers Squibb K.K. Chiyoda‐ku Tokyo Japan; ^20^ Present address: Nobuyuki Hinata, Division of Urology, Graduate School of Biomedical and Health Sciences Hiroshima University Hiroshima Japan

**Keywords:** immunotherapy, nivolumab, observational study, renal cell carcinoma, survival analysis

## Abstract

**Objectives:**

To examine the long‐term effectiveness of nivolumab monotherapy and following subsequent therapies for metastatic renal cell carcinoma (mRCC) in Japanese real‐world settings.

**Methods:**

This was a multicenter, retrospective, observational study, with a 36‐month follow‐up, and conducted in Japanese patients with mRCC who initiated nivolumab monotherapy between 1 Feb 2017 and 31 Oct 2017. Endpoints included overall survival (OS), progression‐free survival (PFS), and objective response rate (ORR).

**Results:**

Of the 208 patients, 36.5% received nivolumab monotherapy as second‐line, 30.8% as third‐line, and 31.7% as fourth‐ or later‐line therapy. By 36 months, 12.0% of patients continued nivolumab monotherapy; 88.0% discontinued, mainly because of disease progression (66.7%). The median (m) OS was not reached irrespective of treatment line, with a 36‐month OS rate of 54.3% (second‐line, 57.4%; third‐line, 52.6%; fourth‐ or later‐line, 52.9%). The ORR was 24.2% and five patients achieved complete response. The OS from first‐line therapy was 8.9 years. In the 95 patients receiving therapy after nivolumab, 87.4% received vascular endothelial growth factor receptor‐tyrosine kinase inhibitors, with mOS and mPFS of 27.4 and 8.1 months, respectively. Irrespective of treatment line, the mOS was not reached in patients with International Metastatic RCC Database Consortium (IMDC) favorable or intermediate risk at mRCC diagnosis.

**Conclusions:**

This 36‐month real‐world follow‐up analysis showed a survival benefit of nivolumab monotherapy for patients with mRCC. The long‐term effectiveness of sequential therapy from first‐line therapy to therapy after nivolumab was also demonstrated. Additionally, nivolumab monotherapy was beneficial for patients with favorable IMDC risk at the time of mRCC diagnosis.

**Abbreviations & Acronyms**
AE: adverse event
BOR: best overall response
CI: confidence interval
DCR: disease control rate
DOR: duration of response
ECOG PS: Eastern Cooperative Oncology Group performance status
HR: hazard ratio
IMDC: International Metastatic RCC Database Consortium
IO: immuno‐oncology
mOS: median overall survival
mPFS: mdian progression‐free survival
mRCC: metastatic renal cell carcinoma
ORR: objective response rate
OS: overall survival
PFS: progression‐free survival
RCC: renal cell carcinoma
TKI: tyrosine kinase inhibitor
VEGFR: vascular endothelial growth factor receptor

## INTRODUCTION

The recent introduction of immuno‐oncology (IO) treatment with agents such as nivolumab has improved the cancer treatment landscape.^1^ Nivolumab was approved in Japan for second‐ or later‐line treatment of patients with metastatic renal cell carcinoma (mRCC) in 2016, based on the CheckMate 025 study.^2^ For first‐line treatment of patients with International Metastatic RCC Database Consortium (IMDC) favorable risk, which is the global standard for determining mRCC treatment, tyrosine kinase inhibitor (TKI) monotherapy or combined IO and TKI treatment (IO + TKI) is recommended.^3^, ^4^, ^5^ If TKI monotherapy is ineffective^5^ or if the treatment is discontinued due to adverse events (AEs), nivolumab monotherapy can be administered as second‐ or later‐line therapy.^3^

Recently, the results of the long‐term follow‐up of CheckMate 025 demonstrated that the median overall survival (mOS) was longer in the Japanese subgroup than in the global population of the study (45.9 and 25.8 months, respectively).^6^, ^7^, ^8^ Longer mOS than the global population of CheckMate 025 has been reported in several Japanese studies evaluating the outcomes of nivolumab monotherapy for mRCC in real‐world settings.^9^, ^10^, ^11^, ^12^ However, these studies provided limited data because of a short follow‐up, a small number of patients, and/or few sites. Therefore, it is important to examine the effectiveness and safety of nivolumab in a larger number of patients with mRCC under real‐world conditions, with a longer follow‐up.

The POST‐NIVO study, a multicenter, retrospective medical record review study conducted in Japan, analyzed nivolumab treatment patterns for patients with mRCC and the effectiveness and safety of nivolumab in a large real‐world population, including patients receiving nivolumab as second‐ or later‐line treatment and those treated with various TKIs as subsequent therapy after discontinuation of nivolumab. The interim analysis reported the effectiveness and safety of nivolumab during the observation period of ≥9 months,^10^ and these results were similar to those of CheckMate 025.^2^ Herein, we report the results of final analysis which showed the effectiveness of nivolumab monotherapy for patients with mRCC in real‐world settings during a 36‐month follow‐up. In addition, we examined the long‐term effectiveness of sequential therapy from first‐line therapy to subsequent therapy after nivolumab discontinuation and the effectiveness of nivolumab according to endpoints including OS by treatment line and IMDC risk at mRCC diagnosis.

## METHODS

### Study design

This was a multicenter, retrospective medical record review study conducted at 17 hospitals in Japan (ClinicalTrials.gov: NCT03568435; UMIN‐CTR: UMIN000033312). The details of the study design have been published.^10^ Briefly, the study was conducted in compliance with the applicable national and international ethical guidelines and the Protection of Personal Information Act. The Ethics Committee of Kindai University Hospital approved the protocol (30‐080) with the Ethics Committee of each participating site. Data were collected from medical records between February 2017 and October 2020. Baseline data were collected between the initial diagnosis of mRCC and immediately before systemic therapy.

### Patients

The study included patients diagnosed with mRCC, based on the Japanese Urological Association guidelines,^3^ who first received nivolumab between 1 February 2017 and 31 October 2017, regardless of the treatment line. Patients could opt out or reject participation. At some sites, patients were required to provide written informed consent by the Ethics Committees. Patients were excluded if aged <20 years or if they had previously participated in any clinical trial of any anticancer agents or in a previous nivolumab regulatory post‐marketing surveillance study (JapicCTI‐184 069).

### Endpoints

The details of endpoints have been published.^10^ Briefly, the endpoints were as follows: treatment patterns of nivolumab including treatment history before and after nivolumab, treatment period, and treatment line. Effectiveness was evaluated by OS, progression‐free survival (PFS), duration of response (DOR), best overall response (BOR), objective response rate (ORR), and disease control rate (DCR) data assessed by investigators per RECIST version 1.1. Other effectiveness endpoints included mOS from the initiation of systemic therapy, mOS by treatment line and IMDC risk at diagnosis, and mOS, mPFS, and ORR of target therapies after nivolumab discontinuation.

### Statistical methods

The statistical methods have been published.^10^ Briefly, patients who met the study criteria were included in the effectiveness analysis. OS, PFS, and DOR were estimated using Kaplan–Meier methodology and hazard ratios (HRs) for OS were estimated using Cox proportional hazards model. Log‐rank test was used to compare the subgroups. Multiplicity was not considered because the study was exploratory. Statistical analyses were conducted using SAS version 9.4 (SAS Institute, Inc.).

## RESULTS

### Patients

The effectiveness set included 208 patients who met the enrollment criteria. Table S1 shows patient demographics and clinical characteristics in the overall population and by nivolumab treatment line. The median ± standard deviation age was 66.5 ± 10.1 years, 76.0% were male, and 57.7% had an Eastern Cooperative Oncology Group performance status (ECOG PS) of 0 or 1. The most common diagnosis was clearcell RCC (76.9%), and the most common metastatic site was lung (74.5%). IMDC risk at mRCC diagnosis and at nivolumab initiation were 20.7% and 10.1% in favorable (no risk factors), 40.9% and 31.7% in intermediate (1 risk factor), 18.3% and 34.6% in intermediate (2 risk factors), 14.4% and 23.1% in poor (≥3 risk factors), and 5.8% and 0.5% in unknown, respectively.

### Treatment patterns

Nivolumab treatment patterns at the 36‐month follow‐up are shown in Table S2. The median number of nivolumab administrations was 12 (range, 1–82), and the median duration of nivolumab was 6.2 months (range, 0.0–42.6). Of the 208 patients, 36.5% received nivolumab as second‐line, 30.8% third‐line, and 31.7% fourth‐ or later‐line therapy. At the 36‐month follow‐up, 12.0% of patients were still on nivolumab monotherapy, and 88.0% had discontinued treatment, mainly because of disease progression (66.7%), followed by AE and/or adverse drug reaction (27.9%), vascular endothelial growth factor receptor‐TKIs (VEGFR‐TKIs) were the most common therapeutic drugs used before (89.9%) and after (39.9%) nivolumab treatment. More than half (113/208; 54.3%) received no treatment other than nivolumab and 77.9% of these (88/113) remained treatment‐free at the 36‐month follow‐up. Table S3 provides treatment patterns after nivolumab in patients who discontinued nivolumab due to disease progression or AEs.

### Effectiveness outcomes

**FIGURE 1:**
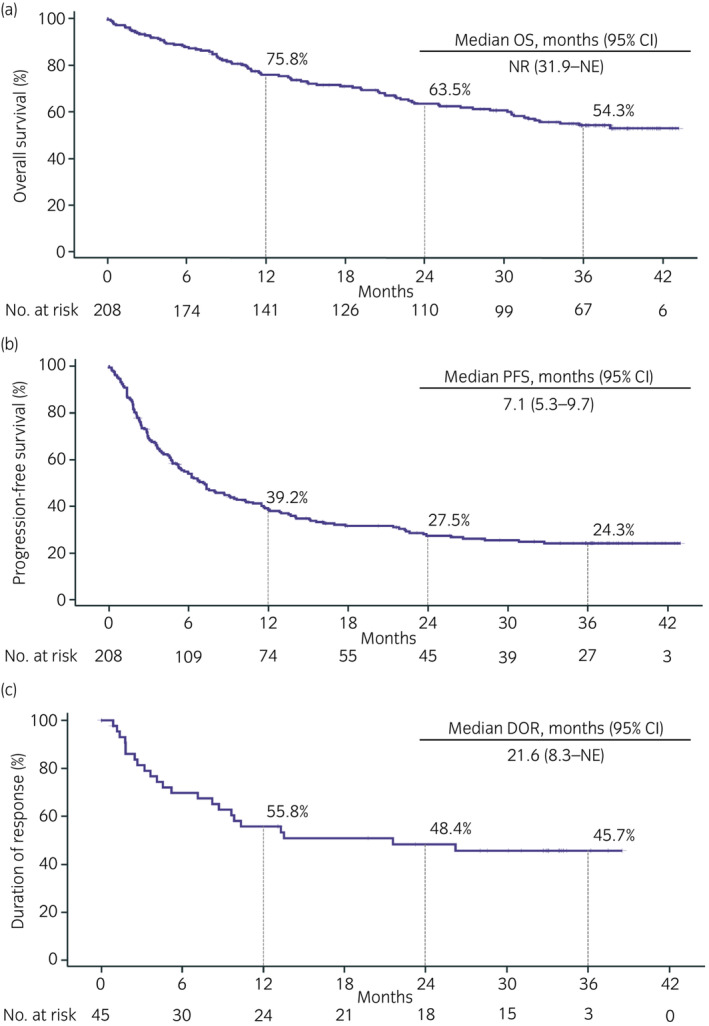
Effectiveness of nivolumab treatment in metastatic renal cell carcinoma. (a) OS, (b) PFS, and (c) DOR. CI, confidence interval; DOR, duration of response; NE, not estimable; NR, not reached; OS, overall survival; PFS, progression‐free survival.

Overall, the mOS was not reached, the 36‐month OS rate was 54.3%, the mPFS was 7.1 (95% confidence interval [CI], 5.3–9.7) months, and the mDOR was 21.6 (95% CI, 8.3–not estimable) months (Figure 1).

At the 36‐month follow‐up, 186 patients (89.4%) were assessed for BOR (Table 1). Five patients (2.7%) had complete response, 40 (21.5%) had partial response, 72 (38.7%) had stable disease and 69 (37.1%) had progressive disease. Twenty‐two patients (10.6%) were not evaluable.

**TABLE 1:** BOR rate, ORR, and DCR

Variable	*N* = 208
Assessment of BOR, *n* (%)	186 (89.4)
BOR^a^	
CR	5 (2.7)
PR	40 (21.5)
SD	72 (38.7)
PD	69 (37.1)
ORR^b^	
*n* (%)	45 (24.2)
95% CI	18.2–31.0
DCR^c^	
*n* (%)	117 (62.9)
95% CI	55.5–69.9

Abbreviations: BOR, best overall response; CI, confidence interval; CR, complete response; DCR, disease control rate; ORR, objective response rate; PD, progressive disease; PR, partial response; RECIST, Response Evaluation Criteria in Solid Tumors; SD, stable disease.

^a^ Calculated from patients who had an assessment of BOR made by investigators, per RECIST version 1.1.

^b^ ORR is the proportion of patients with CR and PR as the BOR.

^c^ DCR is the proportion of patients with CR, PR, or SD as the BOR.

**FIGURE 2:**
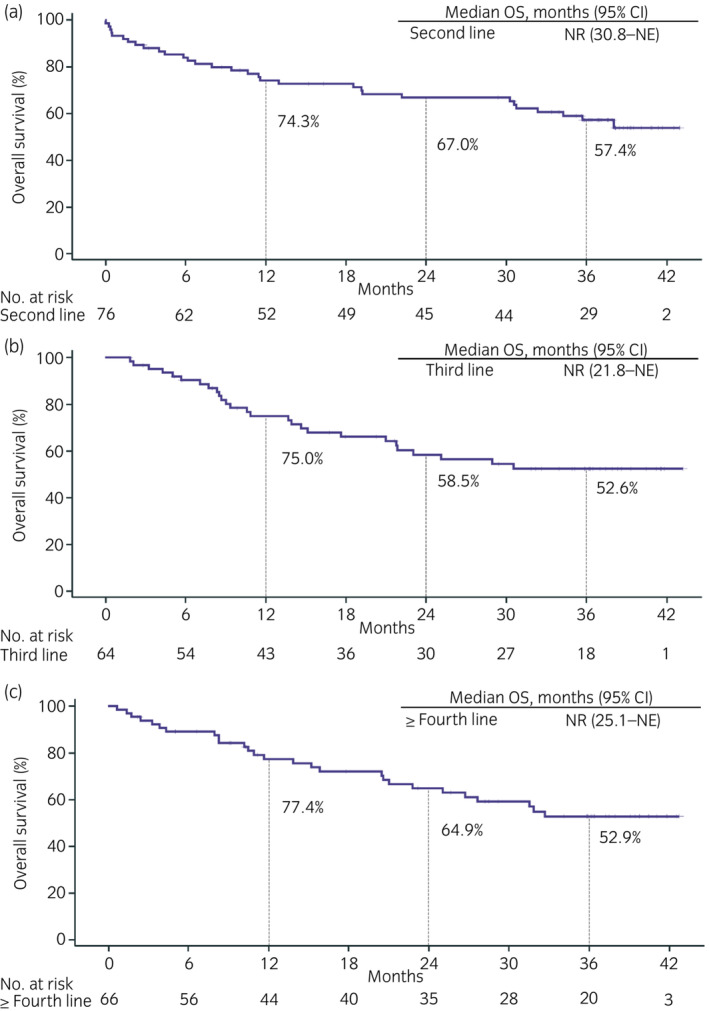
Overall survival (OS) of each line of nivolumab treatment in metastatic renal cell carcinoma. Patients who received nivolumab as (a) second‐line treatment, (b) third‐line treatment, and (c) fourth‐ or later‐line treatment. CI, confidence interval; NE, not estimable; NR, not reached.

**FIGURE 3:**
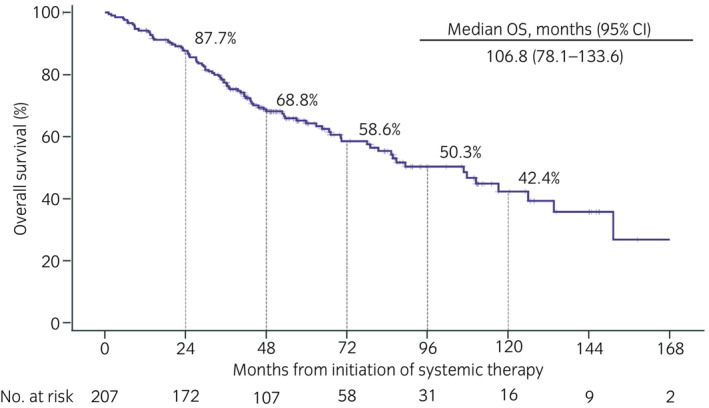
Overall survival (OS) from the initiation of systemic therapy in advanced renal cell carcinoma in this cohort. *N* = 207 (one patient whose start date of systemic therapy was unknown was excluded from this analysis). CI, confidence interval.

The mOS was not reached among patients who received nivolumab monotherapy as second‐, third‐, and fourth‐ or later‐line treatment, with 36‐month OS rates of 57.4%, 52.6%, and 52.9%, respectively (Figure 2). Table S4 shows HRs for OS by subgroups. ECOG PS 0–1 at nivolumab initiation and favorable IMDC risk at mRCC diagnosis and nivolumab initiation were useful prognostic factors (log‐rank *p* < 0.001, <0.001, and <0.001, respectively). For 207 patients, the mOS from the start of first systemic therapy was 106.8 months (95% CI, 78.1–133.6) (Figure 3); one patient was excluded because the start date of systemic therapy was unknown.

**FIGURE 4:**
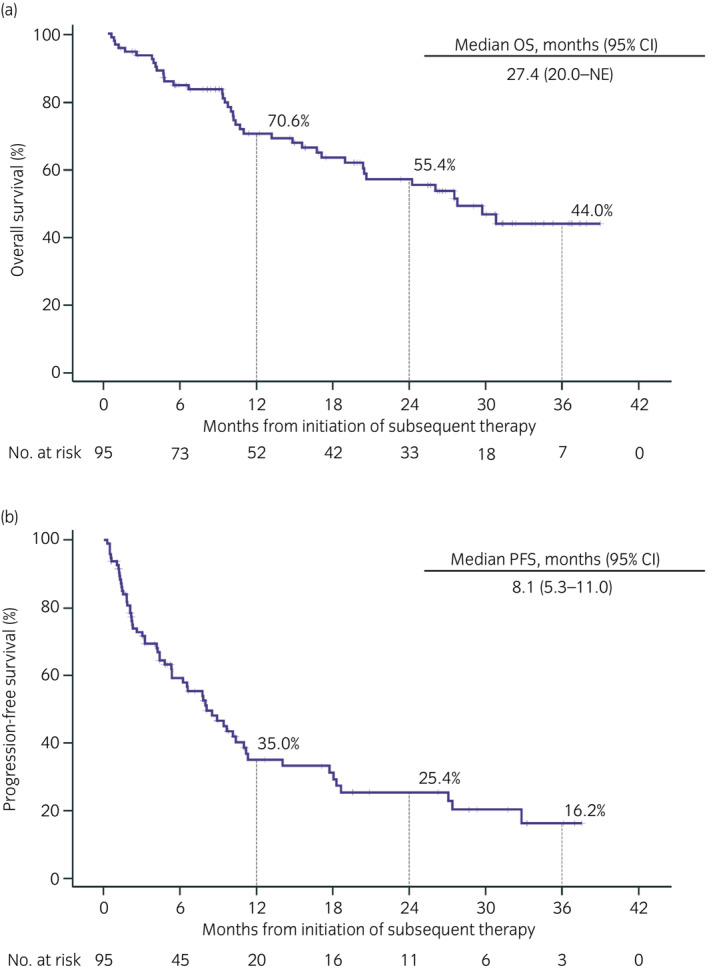
Effectiveness of subsequent therapies after nivolumab discontinuation. (a) OS and (b) PFS. CI, confidence interval; NE, not estimable; OS, overall survival; PFS, progression‐free survival.

A total of 95 patients (45.7%) received treatments after nivolumab discontinuation, with mOS and mPFS of 27.4 months (95% CI, 20.0–not estimable) and 8.1 months (95% CI, 5.3–11.0), respectively (Figure 4). The ORR and DCR of patients who received subsequent therapies are shown in Table 2.

**TABLE 2:** BOR rate, ORR, and DCR of patients who received target therapies after nivolumab discontinuation.

Variable	Patients with post‐nivolumab treatment					
	Overall	Patients with VEGFR‐TKI				
		Overall^a^	Sunitinib	Sorafenib	Axitinib	Pazopanib
	*n* = 95	*n* = 83	*n* = 10	*n* = 6	*n* = 44	*n* = 23
Assessment of BOR						
*n* (%)	70 (73.7)	61 (73.5)	7 (70.0)	4 (66.7)	35 (79.5)	15 (65.2)
BOR^b^						
CR	0 (0.0)	0 (0.0)	0 (0.0)	0 (0.0)	0 (0.0)	0 (0.0)
PR	14 (20.0)	12 (19.7)	3 (42.9)	1 (25.0)	7 (20.0)	1 (6.7)
SD	34 (48.6)	30 (49.2)	0 (0.0)	1 (25.0)	21 (60.0)	8 (53.3)
PD	22 (31.4)	19 (31.1)	4 (57.1)	2 (50.0)	7 (20.0)	6 (40.0)
ORR^c^						
*n* (%)	14 (20.0)	12 (19.7)	3 (42.9)	1 (25.0)	7 (20.0)	1 (6.7)
95% CI	11.4–31.3	10.6–31.8	9.9–81.6	0.6–80.6	8.4–36.9	0.2–31.9
DCR^d^						
*n* (%)	48 (68.6)	42 (68.9)	3 (42.9)	2 (50.0)	28 (80.0)	9 (60.0)
95% CI	56.4–79.1	55.7–80.1	9.9–81.6	6.8–93.2	63.1–91.6	32.3–83.7

Abbreviations: BOR, best overall response; CI, confidence interval; CR, complete response; DCR, disease control rate; ORR, objective response rate; PD, progressive disease; PR, partial response; RECIST, Response Evaluation Criteria in Solid Tumors; SD, stable disease; TKI, tyrosine kinase inhibitor; VEGFR, vascular endothelial growth factor receptor.

^a^ Overall patients with VEGFR‐TKIs as subsequent therapy.

^b^ Calculated from patients who had an assessment of BOR made by investigators, per RECIST version 1.1.

^c^ ORR is the proportion of patients with CR and PR as the BOR.

^d^ DCR is the proportion of patients with CR, PR, or SD as the BOR.

**FIGURE 5:**
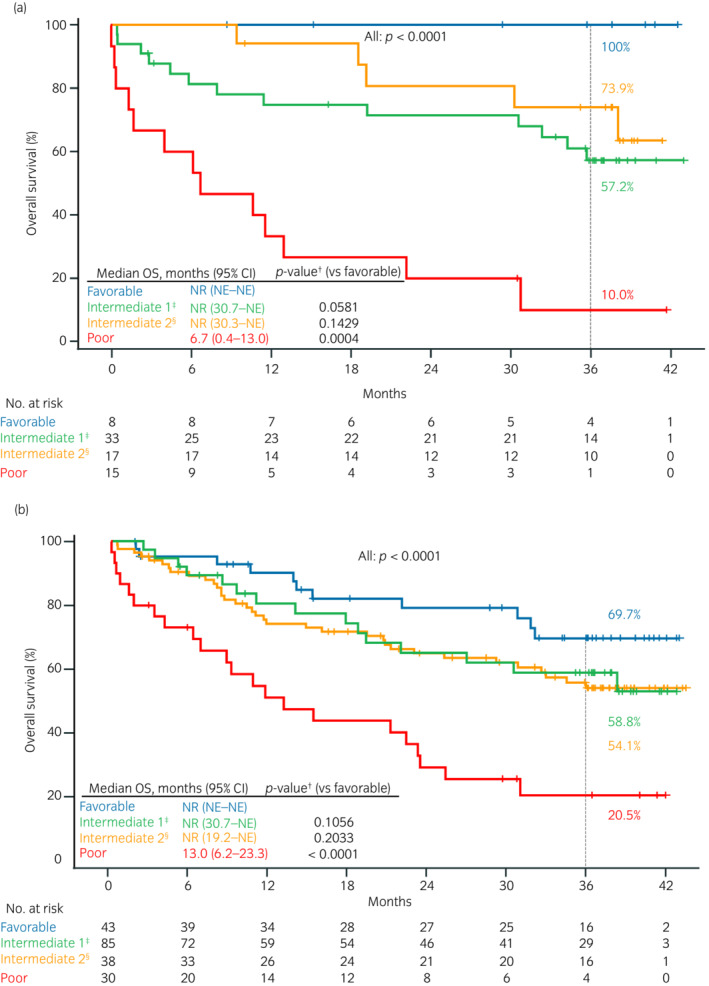
Overall survival (OS) by IMDC risk at diagnosis. (a) Patients who received nivolumab as second‐line treatment and (b) patients who received nivolumab as any line of treatment. ^†^ *p‐*values calculated using log‐rank tests. Multiplicity was not considered because the study was exploratory. ^‡^Intermediate 1 (1 risk factor). ^§^Intermediate 2 (2 risk factors). CI, confidence interval; IMDC, International Metastatic RCC Database Consortium; NE, not estimable; NR, not reached.

Among patients who received nivolumab as second‐line treatment, the mOS was not reached for those with favorable or intermediate IMDC risk at mRCC diagnosis, while those with poor risk had a mOS of 6.7 months (95% CI, 0.4–13.0), with significant differences between patients with favorable and poor risks (*p* = 0.0004) (Figure 5a). Regardless of the treatment line, the mOS was not reached for those with favorable or intermediate risks, while those with poor risk had a mOS of 13.0 months (95% CI, 6.2–23.3), with significant differences between patients with favorable and poor risks (*p* < 0.0001) (Figure 5b). The IMDC risks at mRCC diagnosis by second‐, third‐, and fourth‐ or later‐line treatment are shown in Table S1.

## DISCUSSION

In this 36‐month real‐world data follow‐up analysis, the long‐term effectiveness of sequential therapy including nivolumab monotherapy was examined among patients with mRCC in Japan. Nivolumab monotherapy showed comparable effectiveness to the pivotal CheckMate 025 study. Sequential therapy including nivolumab monotherapy and subsequent TKI monotherapy resulted in favorable prognosis. These findings may support the selection of sequential therapy with IO after TKI in patients with mRCC.

This study included patients with poor prognostic factors (non‐clear cell RCC, 23.1%; brain metastases, 6.3%; Karnofsky Performance Status <80, 12.5%) and >60% of patients were heavily treated (third‐line, 30.8%; fourth‐ or later‐line, 31.7%) and would not have been included in the Japanese subgroup of CheckMate 025.^6^, ^8^ However, the 36‐month OS rate of this study was 54.3%, comparable with that of the global population and Japanese subgroup of CheckMate 025 (39% and 58%, respectively).^6^, ^8^ Furthermore, the mPFS and mDOR (7.1 and 21.6 months, respectively) were numerically longer than those of the global population (4.2 and 18.2 months, respectively, with a minimum follow‐up period of 64 months) or the Japanese subgroup (5.6 and 13.4 months, respectively, with a 36‐month follow‐up) of CheckMate 025.^6^, ^8^ Similarly, for sorafenib, findings in Japanese patients were reported to be more favorable than in the global population.^13^, ^14^, ^15^ The reasons for this are unclear, but may be because of differences in treatment strategies for patients with mRCC in Japan and overseas. Therefore, it is necessary to accumulate evidence of clinical outcomes from treatment strategies in individual countries.^16^ This study provides clinically meaningful information that is important for the consideration of treatment of Japanese patients with mRCC. In a previous Dutch study that retrospectively analyzed nivolumab real‐world data included patients with poor prognostic factors (third‐line, 16%; non‐clear cell RCC, 7%; brain metastases, 11%) and showed mOS and mPFS of 18.7 and 5.6 months, respectively.^17^ The Japanese real‐world data were more favorable than that of the overseas population, with a mOS of over 45.9 months and mPFS of 5.06–10.3 months.^9^, ^11^, ^12^ However, these studies had some limitations in interpreting survival data including short follow‐up durations (≤1 year), a small number of patients (≤100), and/or few sites (≤6).^9^, ^11^, ^12^ Although this study included diverse real‐world patients from 17 sites with a relatively long follow‐up, the results of this study were comparable with those of pivotal trials^6^, ^8^ and other real‐world studies.^9^, ^11^, ^17^

The AFTER I‐O study^18^ retrospectively analyzed Japanese patients with mRCC who received molecular‐targeted therapies after nivolumab and were enrolled in CheckMate 025^2^ or CheckMate 214,^19^ and reported a mOS from first‐line treatment of 70.5 months.^20^ Real‐world data on patients with mRCC reported after the launch of nivolumab in Japan showed a mOS from first‐line treatment of 83.3 months.^21^ The mOS from first‐line treatment in this study (106.8 months; 8.9 years) greatly exceeded that in previous studies,^20^, ^21^ with OS rates of 68.8%, 58.6%, and 50.3% at 48, 72, and 96 months, respectively, despite this study including heavily treated patients and those with poor prognostic factors. A possible reason of this study demonstrated the longer OS from first‐line treatment is that more than half received nivolumab as third‐ or later‐line had favorable ECOG PS or IMDC risk at nivolumab initiation. As the treatment situation for the patients improves, it is expected that more patients with mRCC receive nivolumab treatment as later‐line. Further studies on the effects of nivolumab in later‐line treatment are awaited.

In CheckMate 025, patients with mRCC who had ≥3 total prior systemic treatments were excluded. In the previous Dutch study, third‐ or later‐line nivolumab treatment showed an OS benefit (mOS for second‐line was 18.7 months vs. not reached for third‐ or later‐line therapy).^17^ In the present study, mOS was not reached in any treatment line, but an OS benefit of nivolumab was observed regardless of the treatment line (Figure 2). Additionally, patients treated with nivolumab as third‐ or later‐line therapy had better background factors, including IMDC risk at nivolumab initiation, than those treated with nivolumab as second‐line therapy (Table S1). Furthermore, patients with favorable IMDC risk and ECOG PS at nivolumab initiation had relatively favorable benefit from nivolumab (Table S4). Based on the results of the previous Dutch study^17^ and this study, nivolumab may be potentially effective as later‐line treatment. However, care must be taken to select patients who might fully benefit from such later‐line treatment, such as those with favorable ECOG PS or IMDC risk.

Of the 45.7% of patients in this study receiving treatment immediately after nivolumab, most patients received VEGFR‐TKI (87.4%) with an ORR of 19.7%. Additionally, the mPFS and mOS were 8.1 and 27.4 months, respectively, in line with previous studies.^21^, ^22^, ^23^, ^24^, ^25^ In the recent AFTER I‐O study, 88.5% of patients who received VEGFR‐TKIs and the mPFS and mOS were 8.9 and 29.5 months, respectively.^18^ Both AFTER I‐O^18^ and this study confirmed the effectiveness of post‐nivolumab treatment in clinical settings and showed even greater effectiveness than the AXIS trial (mPFS, 6.7 months with second‐line axitinib).^26^ These data suggest that VEGFR‐TKI treatment after nivolumab is clinically useful.

It is unclear whether IO combination therapy or sequential therapy with IO after TKI treatment improves prognosis in patients with favorable IMDC risk. The IMDC criteria are the most widely used for risk stratification of patients with mRCC and are considered the gold standard for survival prediction.^3^, ^4^, ^5^ According to National Comprehensive Cancer Network guidelines, IO + TKI or TKI monotherapy is the first‐line treatment recommended for patients with favorable IMDC risk.^4^ Recent studies have shown that IO + IO combination therapy improves the prognosis for patients with mRCC^19^, ^27^; however, a meta‐analysis of randomized clinical trials showed no significant difference in OS in patients with favorable IMDC risk for IO + TKI combination therapy compared with TKI monotherapy with sunitinib.^28^ When this study began, TKI monotherapy was the standard therapy. Thus, patients treated with nivolumab as second‐line in this study received IO therapy after TKI monotherapy. Only eight patients in our study had favorable IMDC risk, received nivolumab as second‐line, and had a favorable prognosis (mOS, not reached; 36‐month OS rate, 100%: Figure 5a). In contrast, we observed unfavorable prognoses in patients with poor IMDC risk at mRCC diagnosis (mOS, 6.7 months; 36‐month OS rate, 10.0%), with a significant difference compared with patients with favorable IMDC risk (log‐rank *p* = 0.0004: Figure 5a). This trend was also observed in the overall population regardless of the treatment line, with a significant difference (log‐rank *p* < 0.0001: Figure 5b). As overtreatment with intensive regimens is a concern in current mRCC systemic therapy, it is important to stratify patients according to IMDC risk at mRCC diagnosis (before initiating systemic therapy). Overall, nivolumab as second‐ or later‐line treatment is likely to benefit patients with favorable IMDC risk at mRCC diagnosis who failed first‐line treatment, including TKI monotherapy.

We recognize that our study has some limitations. The main limitations were due to the retrospective, observational design, which may affect the generalizability of our results. Moreover, owing to the observational nature, this study had no control group. Extraction of data from medical records may have limited further analysis because some records might have been incomplete or improperly collected. Additionally, the subsequent use of other treatments in patients who received nivolumab makes it difficult to isolate the independent effect of nivolumab.

In conclusion, this 36‐month real‐world data follow‐up analysis showed a survival benefit of nivolumab monotherapy for patients with mRCC. The long‐term effectiveness of sequential therapy from first‐line treatment to treatment after nivolumab was also demonstrated through the treatment paradigm. In subgroup analyses, consistent effectiveness outcomes were observed regardless of the nivolumab treatment line. Additionally, nivolumab monotherapy after TKI monotherapy was beneficial for patients with favorable IMDC risk at mRCC diagnosis.

## AUTHOR CONTRIBUTIONS

Junji Yonese: investigation, writing—original draft. Nobuyuki Hinata, Satoru Masui, Yasutomo Nakai, Suguru Shirotake, Ario Takeuchi, Teruo Inamoto, Masahiro Nozawa, Kosuke Ueda, Toru Etsunaga, Takahiro Osawa, Motohide Uemura, Go Kimura, Kazuyuki Numakura, Kazutoshi Yamana, Hideaki Miyake, and Satoshi Fukasawa: investigation, writing—review and editing. Naoto Morishima and Hiroaki Ito: funding acquisition, methodology, project administration, visualization, writing—review and editing. Hirotsugu Uemura: methodology, supervision, investigation, visualization, writing—review and editing.

## CONFLICT OF INTEREST STATEMENT

Y.N. received lecture fees from Ono Pharmaceutical and Bristol Myers Squibb. M.N. received lecture fees from Takeda Pharmaceutical and Merck. G.K. received consulting fees from Eisai and lecture fees from Ono Pharmaceutical, Bristol Myers Squibb, Merck Biopharma, Chugai Pharmaceutical, Takeda Pharmaceutical, MSD, and Eisai. K.N. grants or contracts from 2020 the Future Society Business Grant in COVID‐19 era, Akita, Japan, the Suzuki Urology Research Grant, Japan, and 2020 ~ 2022 Grants‐in‐Aid for Scientific Research, the Ministry of Education, Japan, lecture fees from Nihon Medi‐Physics, Pfizer, Merck Biopharma, MSD, Astellas Pharma, Ono Pharmaceutical, Bristol Myers Squibb, Kyowa Kirin, Nippon Kayaku, Takeda Pharmaceutical, and payment for expert testimony from EA Pharma, Ono Pharmaceutical, MSD, Astellas Pharma, and Takeda Pharmaceutical. N.M. is an employee of Ono Pharmaceutical. H.I. is an employee of and owns stock in Bristol Myers Squibb. H.U. received consulting fees from Ono Pharmaceutical, Sanofi, Janssen Pharmaceutical, Bayer, and Pfizer, lecture fees from Bristol Myers Squibb, Pfizer, Janssen Pharmaceutical, Bayer, and MSD, and grants or contracts from Takeda Pharmaceutical, Astellas Pharma, Janssen Pharmaceutical, AstraZeneca, and MSD. J.Y., N.H., S.M., S.S., A.T., T.I., K.U., T.E., T.O., M.U., K.Y., H.M., and S.F. declare that they have no conflicts of interest.

## APPROVAL OF THE RESEARCH PROTOCOL BY AN INSTITUTIONAL REVIEWER BOARD

The Ethics Committee of Kindai University Hospital approved the protocol (30–080) with the Ethics Committee of each participating site.

## INFORMED CONSENT

Patients could opt out or reject participation. At some sites, patients were required to provide written informed consent by the Ethics Committees.

## REGISTRY AND THE REGISTRATION NO. OF THE STUDY/TRIAL

ClinicalTrials.gov: NCT03568435. University Hospital Medical Information Network—Clinical Trial Registration: UMIN000033312.

## ANIMAL STUDIES

Not applicable.

## Supporting information


Appendix S1.
