# Supplementary material for: Real‐world effectiveness of nivolumab and subsequent therapy in Japanese patients with metastatic renal cell carcinoma (POST‐NIVO study): 36‐month follow‐up results of a clinical chart review
Source: Int J Urol. 2023 May 29;30(9):762–71. doi: 10.1111/iju.15206 (PMC11524129; doi:10.1111/iju.15206)
Supplement: Supplementary file 1 — Appendix S1. [file IJU-30-762-s001.docx]

Supplementary Table 1. Patient demographics and clinical characteristics in the overall population and by nivolumab treatment-line

| **Variable** |  | **Overall**  *N* = 208 | Nivolumab treatment-line | | | |
| --- | --- | --- | --- | --- | --- | --- |
|  |  |  | First-line  *n* = 2 | Second-line  *n* = 76 | Third-line  *n* = 64 | Fourth- or later-line  *n* = 66 |
| Age at the start of nivolumab administration (years) | Mean (standard deviation) | 66.5 (10.1) | 61.0 (11.3) | 65.3 (11.0) | 67.7 (10.0) | 66.9 (9.0) |
|  | < 65 | 73 (35.1) | 1 (50.0) | 31 (40.8) | 19 (29.7) | 22 (33.3) |
|  | 65–74 | 92 (44.2) | 1 (50.0) | 32 (42.1) | 28 (43.8) | 31 (47.0) |
|  | ≥ 75 | 43 (20.7) | 0 (0) | 13 (17.1) | 17 (26.6) | 13 (19.7) |
| Sex | Male | 158 (76.0) | 2 (100) | 56 (73.7) | 51 (79.7) | 49 (74.2) |
|  | Female | 50 (24.0) | 0 (0) | 20 (26.3) | 13 (20.3) | 17 (25.8) |
| ECOG PS | 0 | 70 (33.7) | 0 (0) | 23 (30.3) | 21 (32.8) | 26 (39.4) |
|  | 1 | 50 (24.0) | 0 (0) | 19 (25.0) | 15 (23.4) | 16 (24.2) |
|  | 2 | 16 (7.7) | 0 (0) | 3 (3.9) | 9 (14.1) | 4 (6.1) |
|  | 3 or 4 | 10 (4.8) | 0 (0) | 6 (7.9) | 2 (3.1) | 2 (3.0) |
|  | Unknown | 62 (29.8) | 2 (100) | 25 (32.9) | 17 (26.6) | 18 (27.3) |
| Karnofsky Performance Status < 80% at the start of nivolumab administration |  | 26 (12.5) | 0 (0) | 9 (11.8) | 11 (17.2) | 6 (9.1) |
|  | Unknown | 61 (29.3) | 2 (100) | 25 (32.9) | 16 (25.0) | 18 (27.3) |
| Tissue type | Clear | 160 (76.9) | 1 (50.0) | 54 (71.1) | 51 (79.7) | 54 (81.8) |
|  | Non-clear | 48 (23.1) | 1 (50.0) | 22 (28.9) | 13 (20.3) | 12 (18.2) |
| Lung metastasis | Yes | 155 (74.5) | 0 (0) | 50 (65.8) | 47 (73.4) | 58 (87.9) |
| Liver metastasis | Yes | 34 (16.3) | 0 (0) | 12 (15.8) | 9 (14.1) | 13 (19.7) |
| Bone metastasis | Yes | 73 (35.1) | 2 (100) | 24 (31.6) | 23 (35.9) | 24 (36.4) |
| Brain metastasis | Yes | 13 (6.3) | 0 (0) | 5 (6.6) | 3 (4.7) | 5 (7.6) |
| Lymph node metastasis | Yes | 77 (37.0) | 0 (0) | 28 (36.8) | 17 (26.6) | 32 (48.5) |
| Other metastasis | Yes | 90 (43.3) | 0 (0) | 33 (43.4) | 26 (40.6) | 31 (47.0) |
| IMDC risk score at diagnosis of metastatic renal cell carcinoma^†^ | Favorable (no risk factors) | 43 (20.7) | 1 (50.0) | 8 (10.5) | 18 (28.1) | 16 (24.2) |
|  | Intermediate 1 (1 risk factor) | 85 (40.9) | 0 (0) | 33 (43.4) | 24 (37.5) | 28 (42.4) |
|  | Intermediate 2 (2 risk factors) | 38 (18.3) | 0 (0) | 17 (22.4) | 10 (15.6) | 11 (16.7) |
|  | Poor (≥ 3 risk factors) | 30 (14.4) | 1 (50.0) | 15 (19.7) | 10 (15.6) | 4 (6.1) |
|  | Unknown | 12 (5.8) | 0 (0) | 3 (3.9) | 2 (3.1) | 7 (10.6) |
| IMDC risk score at nivolumab treatment^†^ | Favorable (no risk factors) | 21 (10.1) | 1 (50.0) | 4 (5.3) | 10 (15.6) | 6 (9.1) |
|  | Intermediate 1 (1 risk factor) | 66 (31.7) | 0 (0) | 21 (27.6) | 18 (28.1) | 27 (40.9) |
|  | Intermediate 2 (2 risk factors) | 72 (34.6) | 0 (0) | 28 (36.8) | 22 (34.4) | 22 (33.3) |
|  | Poor (≥ 3 risk factors) | 48 (23.1) | 1 (50.0) | 22 (28.9) | 14 (21.9) | 11 (16.7) |
|  | Unknown | 1 (0.5) | 0 (0) | 1 (1.3) | 0 (0.0) | 0 (0.0) |
| Neutrophil-lymphocyte ratio | < 5 | 129 (62.0) | 2 (100) | 40 (75.5) | 40 (78.4) | 47 (83.9) |
|  | ≥ 5 | 33 (15.9) | 0 (0) | 13 (24.5) | 11 (21.6) | 9 (16.1) |
| Lactate dehydrogenase (IU/L) | < 207.8 | 101 (48.6) | 1 (50.0) | 30 (52.6) | 34 (61.8) | 36 (66.7) |
|  | ≥ 207.8 | 67 (32.2) | 1 (50.0) | 27 (47.4) | 21 (38.2) | 18 (33.3) |
| Albumin (g/dL) | < 3.34 | 70 (33.7) | 1 (50.0) | 26 (45.6) | 21 (40.4) | 22 (44.9) |
|  | ≥ 3.34 | 90 (43.3) | 1 (50.0) | 31 (54.4) | 31 (59.6) | 27 (55.1) |
| C-reactive protein (mg/dL) | < 0.8 | 85 (40.9) | 0 (0) | 32 (56.1) | 26 (51.0) | 27 (48.2) |
|  | ≥ 0.8 | 81 (38.9) | 2 (100) | 25 (43.9) | 25 (49.0) | 29 (51.8) |
| eGFR (mL/min/1.73 m^2^) | < 60 | 135 (64.9) | 2 (100) | 46 (79.3) | 42 (76.4) | 45 (81.8) |
|  | ≥ 60 | 35 (16.8) | 0 (0) | 12 (20.7) | 13 (23.6) | 10 (18.2) |

Data are *n* (%) unless otherwise specified.

^†^IMDC risk score was determined by the total number of the following six risk factors that are present: KPS < 80%, time from initial diagnosis to first treatment for metastatic RCC < 1 year, hemoglobin level below the lower limit of the normal range, corrected serum calcium level above the upper limit of the normal range, absolute neutrophil count above the upper limit of the normal range, and platelet count above the upper limit of the normal range.

ECOG PS, Eastern Cooperative Oncology Group performance status; eGFR, estimated glomerular filtration rate; IMDC*,* International Metastatic RCC Database Consortium; RCC, renal cell carcinoma.

Supplementary Table 2. Real-world nivolumab treatment patterns at the 36-month follow-up

|  | *N* = 208 |
| --- | --- |
| Number of doses^†^, median (range) | 12 (1–82) |
| Duration of treatment (months), median (range) | 6.2 (0.0–42.6) |
| Treatment line^‡^, *n* (%) |  |
| First-line | 2 (1.0) |
| Second-line | 76 (36.5) |
| Third-line | 64 (30.8) |
| Fourth- or later-line | 66 (31.7) |
| Ongoing treatment, *n* (%) | 25 (12.0) |
| Discontinuation of nivolumab, *n* (%) | 183 (88.0) |
| Status immediately after nivolumab therapy  Classification, therapeutic drugs, *n* (%) |  |
| VEGFR-TKI | 83 (39.9) |
| mTORi | 11 (5.3) |
| Cytokine | 0 (0) |
| Nivolumab (rechallenge) | 1 (0.5) |
| No treatment after nivolumab therapy | 88 (77.9) |
| Reason for discontinuation of treatment^§^, *n* (%) |  |
| Progression of mRCC | 122 (66.7) |
| Adverse events and/or adverse drug reactions | 51 (27.9) |
| Discontinuation after confirming effectiveness | 2 (1.1) |
| Patient request | 10 (5.5) |
| Death | 10 (5.5) |
| Status immediately before nivolumab therapy^¶^ |  |
| Classification, therapeutic drugs, *n* (%) |  |
| VEGFR-TKI | 187 (89.9) |
| mTORi | 14 (6.7) |
| Cytokine | 2 (1.0) |
| Others | 3 (1.4) |

^†^There were two types of nivolumab dosing schedules, 2-week and 4-week. However, only 2-week dosing was available in 2017.

^‡^All patients received TKI as perioperative treatment.

^§^Multiple answers were allowed.

^¶^Included patients who received nivolumab as second- or later-line therapy.

mTORi, mammalian target of rapamycin inhibitor; mRCC, metastatic renal cell carcinoma; TKI*,* tyrosine kinase inhibitor; VEGFR, vascular endothelial growth factor receptor.

Supplementary Table 3. Real-world treatment patterns after nivolumab treatment by reasons of nivolumab discontinuation

| Nivolumab treatment line | Second-line | | | Third-line | |
| --- | --- | --- | --- | --- | --- |
| Reason for nivolumab discontinuation | Due to disease progression  *n* = 42 | Due to AE  *n* = 24 | Due to disease progression  *n* = 42 | | Due to AE  *n* = 12 |
| Status immediately after nivolumab therapy  Classification, therapeutic drugs, *n* (%) |  |  |  | |  |
| VEGFR-TKI | 26 (61.9) | 8 (33.3) | 21 (50.0) | | 3 (25.0) |
| mTORi | 3 (7.1) | 0 (0) | 6 (14.3) | | 3 (25.0) |
| Cytokine | 0 (0) | 0 (0) | 0 (0) | | 0 (0) |
| Nivolumab (rechallenge) | 0 (0) | 1 (4.2) | 0 (0) | | 0 (0) |
| No treatment after nivolumab therapy | 13 (31.0) | 15 (62.5) | 15 (35.7) | | 6 (50.0) |
| Time to next treatment (days), median (range) | 22 (2–165) | 57 (8–517) | 29 (3–165) | | 118 (15–792) |

AE, adverse event; mTORi, mammalian target of rapamycin inhibitor; mRCC, metastatic renal cell carcinoma; VEGFR-TKI, vascular endothelial growth factor receptor-tyrosine kinase inhibitor.

Supplementary Table 4. Hazard ratios for overall survival by subgroups

|  |  | No. of patients | No. of events | HR (95% CI) | Log-rank P |
| --- | --- | --- | --- | --- | --- |
| Age at the start of nivolumab administration (years) | < 65 | 73 | 31 (42.5) |  | 0.981 |
|  | 65–74 | 92 | 39 (42.4) | 0.96 (0.60–1.54) |  |
|  | ≥ 75 | 43 | 16 (37.2) | 0.95 (0.52–1.74) |  |
| ECOG PS | 0–1 | 120 | 45 (37.5) |  | <0.001 |
|  | ≥2 | 26 | 19 (73.1) | 4.71 (2.70–8.21) |  |
| Tissue type | Clear | 160 | 63 (39.4) |  | 0.068 |
|  | Non-clear | 48 | 23 (47.9) | 1.56 (0.96–2.51) |  |
| IMDC risk score at diagnosis of metastatic renal cell carcinoma^†^ | Favorable (no risk factors) | 8 | 0 (0.0) |  | <0.001 |
|  | Intermediate 1 (1 risk factor) | 33 | 13 (39.4) | Inf |  |
|  | Intermediate 2 (2 risk factors) | 17 | 5 (29.4) | Inf |  |
|  | Poor (≥ 3 risk factors) | 15 | 13 (86.7) | Inf |  |
| IMDC risk score at nivolumab treatment | Favorable (no risk factors) | 21 | 5 (23.8) |  | <0.001 |
|  | Intermediate 1 (1 risk factor) | 66 | 19 (28.8) | 1.18 (0.44–3.17) |  |
|  | Intermediate 2 (2 risk factors) | 72 | 32 (44.4) | 1.96 (0.76–5.04) |  |
|  | Poor (≥ 3 risk factors) | 48 | 30 (62.5) | 4.33 (1.68–11.18) |  |
| Nivolumab treatment line | Second-line | 76 | 31 (40.8) |  | 0.973 |
|  | Third-line | 64 | 27 (42.2) | 1.11 (0.66–1.85) |  |
|  | Fourth- or later-line | 66 | 27 (40.9) | 1.04 (0.62–1.75) |  |

^†^ To examine the impact of IMDC risk at diagnosis in patients who received tyrosine kinase inhibitor monotherapy as first-line therapy and nivolumab monotherapy as second-line therapy, we analyzed data from patients who received nivolumab monotherapy as second-line therapy.

CI, confidence interval; ECOG PS, Eastern Cooperative Oncology Group performance status; HR, hazard ratio; IMDC*,* International Metastatic RCC Database Consortium; Inf, infinity.
